# Randomized phase II exploratory study of prophylactic amifostine in cancer patients who receive radical radiotherapy to the pelvis

**DOI:** 10.1186/1756-9966-29-68

**Published:** 2010-06-10

**Authors:** Konstantinos H Katsanos, Evangelos Briasoulis, Pericles Tsekeris, Anna Batistatou, Maria Bai, Christos Tolis, Antonio Capizzello, Ioannis Panelos, Vasileios Karavasilis, Dimitrios Christodoulou, Epameinondas V Tsianos

**Affiliations:** 1 1st Department of Internal Medicine & Hepato-Gastroenterology Unit, Medical School of Ioannina, Leoforos Stavrou Niarxou, Ioannina, 451 10, Greece; 2 Department of *Medical Oncology, Medical School of Ioannina, Leoforos Stavrou Niarxou, Ioannina, 451 10, Greece; 3 Department of Radiotherapy Medical School of Ioannina, Leoforos Stavrou Niarxou, Ioannina, 451 10, Greece; 4 Department of Pathology, Medical School of Ioannina, Leoforos Stavrou Niarxou, Ioannina, 451 10, Greece

## Abstract

**Background:**

This study aimed to investigate the efficacy of prophylactic amifostine in reducing the risk of severe radiation colitis in cancer patients receiving radical radiotherapy to the pelvis.

**Methods:**

Patients with pelvic tumours referred for radical radiotherapy who consented participation in this trial, were randomly assigned to receive daily amifostine (A) (subcutaneously, 500 mg flat dose) before radiotherapy or radiotherapy alone (R). Sigmoidoscopy and blinded biopsies were scheduled to conduct prior to initiation and following completion of radiotherapy and again 6 to 9 months later. Radiation colitis was assessed by clinical, endoscopic and histolopathological criteria.

**Results:**

A total 44 patients were enrolled in this trial, the majority with rectal (20 patients) and cervical cancer (12 patients) and were assigned 23 in R arm and 21 in the A arm. In total 119 sigmoidoscopies were performed and 18 patients (18/44, 40.9%) were diagnosed with radiation colitis (15 grade 1 and 2, and 3 grade 3 and 4). Of them, 6 patients belonged to the A group (6/21, 28.6%) and 12 to the R group (12/23, 52.2%). Acute and grade IV radiation colitis was only developed in four patients (17.4%) in the R group. Amifostine side effects were mild. Amifostine treated patients were less likely to develop histologically detectable mucosal lesions, which indicate protection from acute mucosal injury.

**Conclusions:**

Amifostine given subcutaneously can lower the risk of acute severe radiation colitis in patients who receive radical radiotherapy to pelvic tumors.

## Background

External beam radiotherapy to the pelvis is related to the development of radiation colitis which is a consequence of radiation-induced mucosal and bowel wall injury. Although in recent years radiation techniques have improved with regard to best dosimetric accuracy, radiation toxicity remains a significant clinical problem resulting in treatment delays, increased patient hospitalisation rates and remarkable short and long-term morbidity [1,2].

Prevention of radiation-induced bowel injury has been the focus of several studies. Among regimens so far investigated one of the best-known radioprotectors is considered to be amifostine. Amifostine is an organic thiophosphate cytoprotective agent known chemically as 2-[(3-aminopropyl) amino] ethanethiol dihydrogen phosphate (ester) [3].

The ability of amifostine to protect normal tissues is attributed to the higher capillary alkaline phosphatase activity, higher pH and better vascularity of normal tissues compared to tumour tissue, resulting in a more rapid generation of the active thiol metabolite and thereby detoxifying the reactive metabolites and scavenging reactive oxygen species generated by radiation [4].

Randomised clinical trials in patients with pelvic malignancies demonstrated that prophylactic amifostine reduces radiation-induced toxicity in lower gastrointestinal tract, pelvic skin and perineal area without compromising the anti-tumor effect [5]. However, results were mainly based on clinical assessments while concomitant endoscopic and histopathologic features of the radiation-induced damage in bowel mucosa were not described [6-10].

The aim of this study was to assess the efficacy of subcutaneous amifostine in preventing radiation colitis in patients irradiated for pelvic neoplasms, by combining clinical, endoscopic and histopathologic data.

## Methods

### Study and Patients

This randomised phase II exploratory clinical trial was activated in May 2001 and conducted in an Academic Hospital [University General Hospital]. The procedures followed were in accordance with the Helsinki Declaration (1964, amended in 1975, 1983, 1989, 1996 and 2000) of the World Medical Association. Institutional review boards and the ethics committee of our University Hospital approved the trial protocol with and patient informed consent. Patients with pelvic malignancies were considered for participation into this trial if they fulfilled a list of eligibility criteria [see below] and signed an informed consent.

Enrolled patients were randomly assigned to receive daily amifostine (subcutaneously, 500 mg flat dose) before radiotherapy (A) or radiotherapy alone (R). Sigmoidoscopy and blinded biopsies were scheduled for all patients prior to initiation of treatment and twice following completion of radiotherapy.

### Study endpoints

The primary study endpoint was to determine the efficacy of amifostine in preventing radiation-induced colitis (RC) by using combined clinical, endoscopic and histopathologic data from patients irradiated to the pelvis.

The secondary endpoints of the study were the assessment of agreement between clinical, endoscopic and histopathologic data during radiotherapy and post-radiotherapy period and the evaluation of amifostine-related toxicity.

### Eligibility criteria

The study enrolled patients with primary pelvic or metastatic to the pelvis malignancies who were referred for adjuvant, radical or palliative radiotherapy but not for re-irradiation. All patients recruited in the study were older than 18 years, had a World Health Organization (WHO) performance status 0-2 and a life expectancy of more than 6 months. Pregnant or lactating women, patients with severe infections or severe psychiatric or neurologic illnesses were excluded. Patients with decreased hematologic reserves, with major organ failure, severe electrolyte or metabolic abnormalities were also excluded. In patients with haemoglobin levels below 11 g/dl before radiotherapy, subcutaneous erythropoietin was administered. Patients with hypertension controlled with medication were eligible for amifostine administration. Patients with asymptomatic low blood pressure were included. Patients with symptomatic hypotension were excluded. Patients with a previous history of chronic colitis, non-specific proctitis, ulcerative colitis, diverticular disease or those who were on treatment with non-steroidal anti-inflammatory drugs were excluded.

### Basic assessments and randomisation

Pre-radiotherapy assessment included a detailed medical history, complete physical examination, peripheral blood count and biochemistry, electrocardiogram, chest X-ray, computed tomography or magnetic resonance imaging of the abdomen and pelvis, bone scintigraphy -when indicated- and flexible sigmoidoscopy with bowel biopsies from areas included within the radiation fields.

All patients were randomised 1:1 to receive subcutaneous amifostine (*Ethyol, Schering Plough S.A*) immediately before each fraction of radiotherapy (Group A) or radiotherapy alone (Group R).

### Radiotherapy modifications

All patients but one received radical or postoperative external beam radiotherapy by a linear accelerator (6 MV) and one patient was treated using a Cobalt-60 unit. Four parallel opposed fields - anteroposterior, posteroanterior and two laterals- were applied (box technique). The median daily radiation dose was 1.9 Gy. All fields were treated every day (5 fractions/week) and the mean number of fractions per patient was 28 (range 23-36 fractions).

Reasons for treatment discontinuation were disease progression during treatment, severe or life threatening radiation toxicity, patient decision to stop treatment, poor patient compliance or systemic reactions due to amifostine use. All patients with any sign of severe toxicity not responding to standard measures discontinued radiotherapy.

### Amifostine administration

Patients randomised to the A group (Amifostine plus Radiotherapy) were adequately hydrated and pre-treated with antiemetics 1-2 hours prior to the administration of amifostine. Amifostine was given subcutaneously at a flat dose of 500 mg. Amifostine injection was repeated daily (5 days/week), 20-30 minutes before radiotherapy.

### Endoscopic surveillance and follow-up

All patients in both groups (A and R) were planned to undergo three endoscopies (sigmoidoscopies, up to the splenic flexure). The first sigmoidoscopy would be performed before the initiation of radiotherapy, the second after the completion of radiotherapy (approximately 40 days after the first) and the third at least six months after the end of radiotherapy.

Diagnosis of radiation colitis (RC) was based on patients' symptoms, laboratory tests, endoscopic and histological findings. Biopsy specimens from each patient consisted of at least 3 samples of large bowel mucosa, taken blindly from the region included in the radiation field every 10 cm, or from areas that appeared to be affected (at least one sample), as well as from normal-appearing mucosa (at least one sample). The same gastroenterologist, who was blinded to the patient treatment arm, assessed in each endoscopy the extent and the degree of colonic mucosal damage.

Radiation toxicity to the bowel was assessed using the RTOG/EORTC late radiation morbidity scale for large intestine as the only validated currently available scale [11]. Patients diagnosed with radiation colitis (RC) were divided into early (acute) onset RC (symptoms of RC within 6 months after completion of radiotherapy) and late onset RC (RC occurring later than 6 months after the end of radiotherapy).

### Assessment of response to radiotherapy

We monitored patients during daily radiotherapy sessions and also during post-radiotherapy follow up. Response assessment to radiotherapy was assessed by means of computed tomography and endoscopies. In addition, WHO performance status, bowel overall function and daily movements, blood pressure and body weight were also monitored.

### Evaluation of toxicity

During radiotherapy and on a weekly basis, clinical examination and signs of toxicity were recorded according to Common Toxicity Criteria (*CTC, version 2.0*). Amifostine toxicity was also assessed by the CTC criteria. After the end of radiotherapy and every three months for the first two years and then every six months for the next years, clinical examination and evaluation of toxicity were also planned.

### Histopathological study

Bowel mucosa biopsies were fixed in 4% buffered formalin, embedded in paraffin and cut in 5 μm sections. For histological evaluation the sections were stained with the standard haematoxylin-eosin (H&E) stain. Furthermore, immunostaining was performed by the labeled straptavidin-avidin-biotin method (LSAB Kit, Dako SA, Glostrup, Denmark) using the monoclonal antibody directed against active caspase 3 (dilution 1:500; clone C92-605, Pharmigen, San Diego, CA), as previously described [12].

### Evaluation of Haematoxylin-eosin (H&E) staining

Since there are no general and precisely defined criteria for histologic diagnosis and grading of radiation colitis our histologic reports were based on relevant studies and textbooks [13,14]. According to these colitis lesions were graded as absent (-), mild (+) and moderate to severe (++/+++). Histologic features of colitis included presence of increased inflammatory infiltration of the lamina propria (estimation of proportion of neutrophils, eosinophils, lymphocytes and plasma cells, as well as the presence of muciphages-foamy cells), presence of erosions or ulcers, absence of viable crypts and presence of cryptitis (inflammatory cells permeating the crypt epithelium and destroying crypts) and crypt abscesses (cellular cell irregularities, cytoplasmic vacuolation, nuclear abnormalities, increased apoptotic bodies), architectural crypt distortion (crypt branching and shortening, crypt disarray-slight distortion with widening, atrophy) presence of fibrosis of the lamina propria, vascular changes (telangiectasia, endothelial degeneration, platelet thrombi formation).

### Evaluation of immunostaining

The number of active caspase 3 positive epithelial cells, within the surface epithelium as well as within the crypts, was recorded by using the ×40 objective lens. Since the tissue contained in the biopsies was limited, the whole biopsy area was evaluated in all cases. The apoptotic index (AI) was expressed as the number of active caspase 3-positive epithelial cells per 10 high power fields. Tissue sections were examined independently by two of the authors who were blinded to the treatment group and to the sigmoidoscopy findings. Discrepancies were resolved at the discussion microscope.

### Statistical Analysis

The sample size in the study was set for logistic reasons to 40 patients; minimum 20 patients per treatment arm. Continuous variables were described as means ± SD when were normally distributed or as median with maximal and minimal range for observations not normally distributed. Comparison between groups was performed using ANOVA and Student's t-test. X^2 ^analysis was used when comparing frequencies. A p value < 0.05 (two-tailed) was considered to be significant. For all calculations we used the SPSS 12.0 working package (SPSS Inc., Chicago, IL).

## Results

A total of 44 patients (23 females, 21 males) with a median age of 63 years (range 35-79 years) were enrolled in this trial. Of them, 20 had rectal cancer, 12 cervical cancer, 5 prostate cancer, 3 urinary bladder cancer, 2 endometrial cancer and 2 sarcomas of the pelvis. Twenty-one patients were randomised to receive amifostine prior to radiotherapy (group A) and 23 patients received only radiotherapy (group R).

Radical radiotherapy was administered in 24 patients. Adjuvant radiotherapy was administered in 20 patients (15 with rectal cancer, 3 with cervical cancer, and 2 with pelvic sarcoma). Patient characteristics are summarized in Table 1.

**Table 1:** Demographics and study characteristics in cancer patients receiving external pelvic radiotherapy with or without amifostine prophylaxis.

	Total	A*	R**
**No of patients treated**	44	21	23

**Gender:**			
Female	23	15	8
Male	21	5	16

**Age:**			
Median (range)	63(34-79)	59	62

**Tumor types:**			
Rectal	20	7	13
Cervical	12	8	4
Prostate	5	2	3
Bladder	3	1	3
Endometrial	2	2	-
Sarcoma	2	-	2

**Mean radiation dose (Gy):**		50.4	50.2

*A = Amifostine

**R = Radiotherapy alone

### Radiotherapy dose

The mean total radiation dose was 50.4 Gy for the amifostine plus radiation group (A) and 50.2 Gy for the radiotherapy alone group (R). Nine females with cervical cancer received additional brachytherapy with median total dose of 24 Gy. There was no significant difference between the total RT dose in patients diagnosed with or without radiation colitis (50.3 Gy in both groups, p > 0.5).

### Radiotherapy delays and amifostine toxicity

All patients completed radiotherapy as planned. Two patients in the A group (1 patient with cervical and 1 patient with prostate cancer) temporarily interrupted radiotherapy on weeks 2 and 3 respectively due to side effects unrelated to amifostine (neutropenia grade 3). Radiotherapy was restarted in both of them 3 weeks later and was completed uneventfully.

No dose adjustment of amifostine was made for toxicity. Amifostine-related side effects occurred in 4 out of 21 patients (19%) and were mild. Two patients developed local erythema and pruritus at the site of amifostine subcutaneous injection (grade 1) and 2 patients had nausea or/and vomiting grade 1 and 2.

Within a median follow up time of 24 months, one patient with bladder cancer and one patient with rectal cancer operated due to local relapse after radiotherapy and 5 patients (5/44 = 11.4%) died. None of deaths was associated to radiation colitis or amifostine but was solely attributed to disease progression.

### Endoscopic findings

A total of 119 sigmoidoscopies were performed. All patients had a baseline sigmoidoscopy and at least one follow-up endoscopy as planned (median 2.7 endoscopies per patient). There were no significant differences between the two groups (A vs R) regarding patient age, time of follow-up or cumulative number of endoscopies [in detail, 59 vs 62 years of age, 24.5 vs 23.5 months of follow up, 58 vs 61endoscopies].

**Figure 1:**
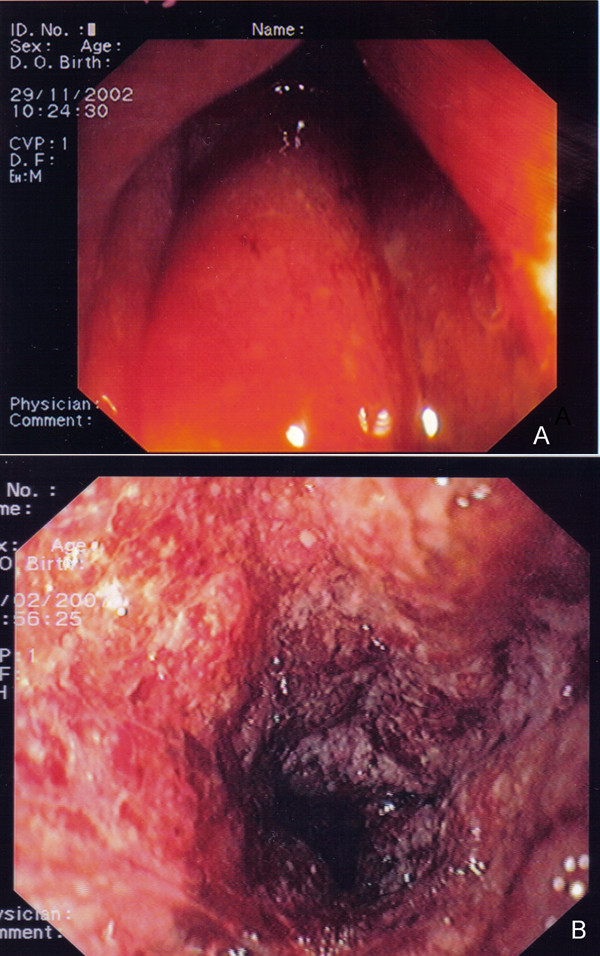
**A. Congested rectal mucosa with diffuse erythema in a case of grade I radiation colitis (RTOG/EORTC late radiation morbidity scale for large intestine)**. **B**. Ulcerated rectal mucosa with diffuse erythema, mucous and intermittent bleeding in a case of grade II radiation colitis (RTOG/EORTC late radiation morbidity scale for large intestine).

Eighteen out of 44 patients (40.9%) were diagnosed with radiation colitis (RC). Of these 18 patients, 6 were in the A group (6/21 patients = 28.6%) and 12 in the R group (12/23 patients = 52.2%) [p = 0.29]. The endoscopic findings and grading of RC are listed in Table 2. Sigmoidoscopic findings ranged from minor signs of inflammation to more prominent signs of bowel mucosa injury (Figures 1A-B).

**Table 2:** Endoscopic findings and grading of radiation colitis in cancer patients receiving external pelvic radiotherapy with or without amifostine prophylaxis.

	A + R (N = 21)		R (N = 23)	
Endoscopically rated colitis	Acute	Late	Acute	Late

Grade 1	-	-	-	2

Grade 2	-	6	2	6

Grade 3	-	1	1	-

Grade 4	-	-	1	-

Totals (%)	- (0%)^+^	7 (28,6%)	4 (17,4%)^+^	8 (34,8%)

*A = Amifostine

**R = Radiotherapy

^+ ^*p = 0.05*

Four patients (17.4%) in the R group developed acute colitis and two of them required hospitalization. By contrast none of the patients in the A+R group developed acute colitis [17.4% vs 0%, p = 0.05].

### Histopathological findings

#### Haematoxylin-eosin staining

**Figure 2:**
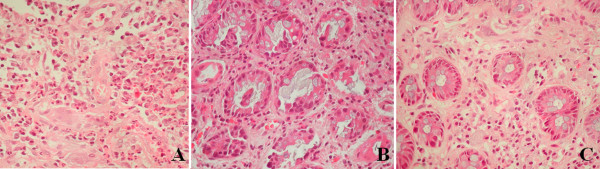
**Histopathological findings of radiation-induced colitis**. **A**. Acute injury, characterized by ulceration, absence of viable crypts, diffuse infiltration by polymorphonuclear leucocytes, and prominent capillaries lined by plump endothelial cells (H + E × 400). **B**. Early regenerative changes, characterized by absence of ulceration, considerably less acute inflammation, infiltration by plasma cells and lymphocytes, presence of viable crypts with disarray, absence of cryptitis or acute epithelial damage (H+E × 400). **C**. Late regenerative changes, characterized by absence of acute inflammation, mild diffuse infiltration by plama cells and lymphocytes, architectural crypt distortion, with reduced crypts, crypt branching and shortening as well as moderate/severe fibrosis of the lamina propria (H + E × 400).

**Figure 3:**
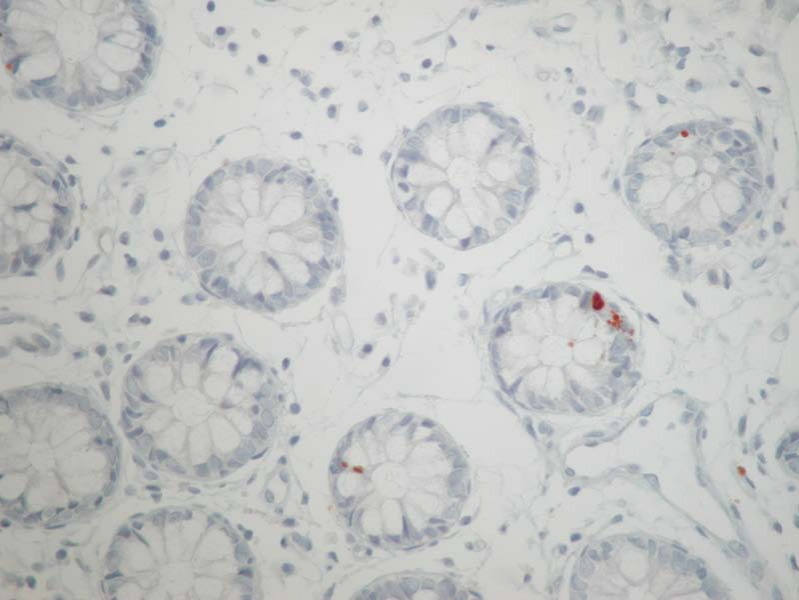
**Immunohistochemical expression of active caspase 3 in apoptotic epithelial cells**.

Based on the histologic features noted with the haematoxylin-eosin stain, cases were allocated to one of the following four groups: 1) no changes, when no changes were noted in bowel mucosa; 2) acute injury, characterized by the presence of ulceration and diffuse infiltration by polymorphonuclear leucocytes as well as eosinophil granulocytes, the near absence of viable crypts, the presence of cryptitis and other damage in the surviving epithelium, endothelial degeneration and platelet thrombi (Figure 2A); 3) early regenerative changes, characterized by absence of ulceration, considerably less acute inflammation but noteworthy infiltration by eosinophil granulocytes, as well as plasma cells, lymphocytes and muciphages, presence of viable crypts with disarray, absence of cryptitis or acute epithelial damage, some endothelial degeneration and telangiectasia as well as mild fibrosis of the lamina propria (Figure 2B) and 4) late regenerative changes, characterized by minimal (if any) acute inflammation, mild diffuse infiltration by plama cells and lymphocytes, consistent presence of muciphages, architectural crypt distortion, with reduced crypts, crypt branching and shortening as well as moderate/severe fibrosis of the lamina propria (Figure 2C). The presence of eosinophils in the lamina propria, particularly on the acute and early regenerative phase was noted in almost all specimens. However, in contrast with the radiation colitis induced by pre-operative irradiation no "eosinophil crypt abscesses" was observed, even in acute injury.

In a minority of our patients an acute mucosal injury, was diagnosed histologically. More specifically, of all patients administered amifostine, none exhibited acute mucosal injury, regardless of the biopsy timing (early or late). Furthermore, of the eight patients receiving amifostine and undergoing early biopsies, four (50%) exhibited early regenerative changes; two (25%) late regenerative changes and two (25%) had no abnormal histological findings. Of the fourteen patients receiving amifostine and undergoing late biopsies, three (21.4%) showed early regenerative changes, nine (64.3%) late regenerative changes and two (14.3%) had no abnormal histological findings. Acute mucosal injury was histologically characterized in three patients who did not receive amifostine; in two out of the seven (28.6%) patients with early biopsies and one out of the fifteen patients with late biopsies (6.7%).

Furthermore, in arm R, early biopsies early regenerative changes in two (28.6%) and late regenerative changes in two (28.6%) patients. In the same group, late biopsies revealed early regenerative changes in five (33.3%) and late regenerative changes in eight (53.3%) patients. The differences between the groups were not statistically significant.

#### Immunohistochemistry

The apoptotic index (AI) was calculated in bowel specimens from both groups (A and R) and was analysed in relation to the timing of radiotherapy (AI1 = biopsy before the initiation of radiotherapy, AI2 = biopsy after the completion of radiotherapy and AP3 = biopsy at least six months after the end of radiotherapy). In the A group of patients the AI1, AI2 and AI3 were [mean ± SD] 1.0 ± 0.6, 1.1 ± 0.7 and 1.4 ± 0.8 and in the R group of patients the AI1, AI2, AI3 were 1.0 ± 0.6, 1.3 ± 1.0 and 0.9 ± 0.3 respectively [Figure 3]. No significant differences were observed for the AI1, AI2 and AI3 between the two patient groups.

#### Concordance of endoscopic and histopathological findings

The concordance between histologically defined radiation colitis and endoscopic findings was rather poor with endoscopy findings underestimating bowel mucosal injury. Characteristically, in patients with endoscopically mild to moderate colitis (EORTC/RTOG grade 1-2) the corresponding large bowel mucosa histologic changes were disproportionally pronounced.

#### Radiation colitis management

All cases of RC were manageable. In cases of mild to moderate RC (grade I and II), patients were treated on outpatient basis. For more severe symptoms (grade III and IV) hospitalisation was necessary for 10-15 days. Mild and moderate RC cases were treated with corticosteroid and mesalamine enemas administered twice daily for a period of 10-20 days according to clinical response.

## Discussion

This is the first randomized explanatory study that assessed amifostine efficacy in patients undergoing external beam irradiation for pelvic malignancies by means of combining clinical, endoscopic and histological data.

Patients on prophylactic subcutaneous amifostine developed less acute RC compared to patients who did not receive amifostine prophylaxis, yet the small size of this study did not allow us to reach to statistically significant findings. However, acute RC and grade IV radiation colitis did not occur in the amifostine arm but only in four patients (17.4%) who did not receive amifostine prophylaxis (arm R). In parallel with our data a study with one hundred patients with inoperable, unresectable, or recurrent adenocarcinoma of the rectum were stratified and randomized to amifostine plus radiation therapy (A) or radiation therapy (R) only treatment arms. According to this study, the administration of amifostine concomitant to radiation for advanced rectal cancer, was reported to significantly reduce acute and late pelvic radiation toxicity [15,16].

Furthermore, several studies have also shown a radiation protective function of amifostine to perineal, skin, bladder, and bowel mucosa in patients irradiated for pelvic area malignancies [17-31]. Overall, there is accumulating data demonstrating that amifostine may protect from acute and late onset colitis and well-designed short and long-term protection protocols may prove of great importance.

Reporting and assessing toxicity is important in all oncological trials. This study confirmed what others have already shown that subcutaneous amifostine at 500 mg is well tolerated [5].

Pathologists are familiar with delayed colitis, which develops months to years after pelvic radiotherapy for rectal, gynecologic, or bladder cancers but grading acute radiation injury to bowel mucosa represents an unaddressed issue. Differential diagnosis of acute or late onset radiation colitis is broad. It is noteworthy that the presence of nuclear abnormalities in acute radiation colitis may mimic epithelial dysplasia in ulcerative colitis [32].

In contrast to reported observation of eosinophilic crypt abscesses in irradiated bowel mucosa in cancer patients who received pre-operative irradiation, such findings were not observed in our patients, even in cases with an acute RC. Another study [18] had systematically characterized acute radiation colitis in patients treated with short-term preoperative radiotherapy for rectal cancer. However, due to the nature of the material examined (surgical resection specimens) in that study no correlation with endoscopical findings was made. In addition, findings analyzed were representing areas from peritumoral colonic mucosa, which conceivably could be affected by the adjacent tumor. Other investigators have addressed interesting issues regarding RC pathogenesis, besides morphology, and have reported that transient aberrant expression of P-cadherin may be associated with proctitis [33].

In an interesting study [34], also supportive of the prophylactic role of amifostine, radiation-induced acute rectal toxicity was evaluated by using three different toxicity scales: WHO scale, EORTC/RTOG toxicity criteria, and a modified toxicity scale. In the present study we have used precisely defined criteria for grading of acute and also of late radiation colitis, based on published reports and textbooks, and thus we were able to semiquantitavely compare histologic changes and endoscopy between groups. From the histologic data it is evident that patients receiving amifostine are less likely to develop histologically detectable mucosal changes Furthermore, the administration of amifostine appears to protect patients from acute mucosal injury. We have further extended our histopathologic study by examining the immunohistochemical expression of active caspase-3. Immunohistochemical expression of active caspace 3 in cells is a valuable means of detection of apoptosis induced by a wide variety of apoptotic signal [12].

We detected active caspase-3 in all biopsy specimens, early or late, with or without amifostine, even in pre-radiation biopsies. However, significant differences between treatent arms were not detected. This is probably due, at least in part, to drop-out of the epithelium in the acute injury phase, were the apoptotic index (AI) should be the highest.

It is of interest that in both patient groups in this study the correlation of histopathologically defined phases of radiation-induced colitis and endoscopic findings was poor. Consistent with these data is another study [35], which failed to show a correlation between histopathological findings and clinical status of patients with colon cancer treated pre-operatively with irradiation. The observations of this study indicate that acute radiation colitis may remain clinically silent and resolve spontaneously within a few weeks after irradiation. Given the increasing acceptance of short-term preoperative irradiation protocols for rectal cancer, pathologists should be aware of the rather characteristic histopathologic findings of acute radiation colitis and avoid unnecessary concern of clinicians.

## Conclusions

In conclusion, this is one of the first studies to assess the efficacy of prophylactic amifostine efficacy by using clinical, endoscopic and histologic assessment in patients receiving radical radiotherapy to pelvic tumors. Subcutaneous amifostine prophylactic was safe and seemed to provide protection to the development of severe and acute radiation colitis. Larger studies and longer follow up is needed to confirm and evaluate the long-term protective function of amifostine.

The poor concordance of endoscopic and histologic findings undercores the need for a global assessment of radiation-induced bowel injury by clinical, endoscopic, and histological means.

## List of abbreviations used in the text

**RC**: radiation colitis; **WHO**: World Health Organization; **RTOG/EORTC**: Radiation Therapy Oncology Group/European Organization for Research and Treatment of Cancer; **CTC**: Common toxicity criteria (*version 2.0*)

## Competing interests

The authors declare that they have no competing interests.

## Authors' contributions

KHK coordinated the study and drafted the manuscript, EB conceived the study and participated in its design and coordination and helped to draft the manuscript, PT conceived the study and participated in its design and coordination, AB carried out the histology and immunohistochemical studies and helped to draft the manuscript, MB carried out the histology and immunohistochemical studies, CT and helped to draft the manuscript, AC participated in its design and coordination, IP carried out the histology and immunohistochemical studies, DC participated in its design and coordination, EVT conceived the study and participated in its design and coordination.

All authors read and approved the final manuscript.
